# Correlation between TyG index and coronary atherosclerosis assessed by CCTA in elderly male patients: a cross-sectional study

**DOI:** 10.1186/s13098-023-01145-3

**Published:** 2023-08-24

**Authors:** Xiaona wang, Xinqiang Ji, Jianhui yu, Fan wang

**Affiliations:** 1 Department of Cardiology, The Second Medical Center & National Clinical Research Center for Geriatric Diseases, Chinese PLA General Hospital, Beijing, 100853 China; 2 Chinese PLA Medical School, Beijing, 100853 China

**Keywords:** TyG index, Coronary atherosclerosis plaque burden, CCTA, Leiden score

## Abstract

**Background:**

Age is a major risk factor associated with the complexity of coronary artery disease (CAD), and the prognosis of elderly patients with coronary heart disease is relatively poor. Metabolic disturbances are prevalent in the elderly population and contribute to CAD morbidity and mortality. This study aimed to investigate the relationship between the triglyceride-glucose (TyG) index and total coronary atherosclerotic burden assessed non-invasively by Coronary Computed Tomography Angiogram (CCTA) in the elderly population.

**Methods:**

This retrospective cross-sectional study involved 427 patients who underwent CCTA. The patients were divided into two groups based on their Leiden score: ≥5 and < 5. Comparisons between groups were conducted using t-tests or Mann-Whitney U tests for continuous variables and chi-square tests for categorical variables. The correlation between TyG and Leiden score was assessed using Spearman’s rank correlation coefficient. Univariable and multivariable logistic regression analyses were performed to assess the role of TyG in atherosclerosis risk, using clinical variables previously shown to independently predict a high Leiden score.

**Results:**

The levels of age and HbA1c% were significantly higher in patients with Leiden score ≥ 5. Patients with Leiden score ≥ 5 showed no significant difference in TyG index compared to those with Leiden score < 5. Pearson correlation analysis showed that HbA1c% (r = 0.44, p < 0.01), age (r = 0.34, p < 0.01), and FBG (r = 0.15, p = 0.01) were positively correlated with Leiden score, while TyG index had no correlation with Leiden score (r = 0.05, p = 0.42). Multiple linear regression analysis showed that HbA1c% (β = 2.92, 95%CI: 2.25–3.56, P < 0.01) was positively correlated with Leiden score, while TyG index had no correlation with Leiden score (β = 0.73, 95%CI: -3.27-4.72, P < 0.01). HbA1c% was found to be an influential factor for obstructive CVD (β = 1.86, 95%CI: 1.50–2.29, P < 0.01), while TyG index was not an independent risk factor for obstructive CVD (β = 0.39, 95%CI: 0.12–1.32, P = 0.13).

**Conclusion:**

The TyG index did not show any significant correlation with the Leiden score and obstructive CVD as a risk factor in elderly male population. On the other hand, HbA1c% was identified as an influential factor for both the Leiden score and obstructive CVD.

## Introduction

In the last decade, China has experienced rapid aging and has become the largest aging country globally [1]. Life expectancy has also increased from 74.8 years to 78.2 years, and it is projected to surpass 80 years for both sexes by 2040 [2]. Aging is accompanied by changes in vascular structure and function, particularly in the large arteries [3, 4]. Age is a significant risk factor associated with complex coronary artery disease, and the prognosis of elderly patients with coronary heart disease is relatively poor [5]. Recently, Coronary computed tomography angiography (CCTA) plaque burden scores, including the Leiden score, have been adopted and validated for long-term cardiac event prediction [6, 7]. Moreover, metabolic disturbances are more prevalent in the elderly population and contribute to cardiovascular morbidity and mortality [8]. The triglyceride-glucose (TyG) index, a reliable surrogate marker of insulin resistance (IR), is strongly associated with coronary atherosclerosis and has clinical significance in risk stratification and prognosis evaluation of coronary artery disease [9–13]. This study aims to determine the relationship between TyG and total coronary atherosclerotic burden assessed non-invasively by CCTA in the elderly population.

## Method

### Study population

The study population included male patients aged 75 years and above who were diagnosed with coronary artery disease (CAD) and had undergone CCTA at the Second Center of PLA General Hospital from January 2017 to September 2020.

Inclusion criteria: Subjects aged 18 and above who had examined triglyceride and glucose within one month after CCTA were included. Exclusion criteria: Subjects with revascularization, previous myocardial infarction or structural heart disease were excluded.

A total of 427 patients who met the enrollment criteria were ultimately included in the present analyses. The PLA General Hospital ethics committee granted ethical approval.

### CCTA acquisition and image analysis

All CT examinations were performed on a dual-source CT scanner (Somatom Definition Flash, Siemens, Florsheim, Germany), and data reconstruction was performed using multi-planar reconstruction and maximum density projection on a dedicated workstation (Syngo.via, Symens, Florsheim, Germany). A 17-segment mode was adopted to assess coronary anatomy. Each coronary plaque was visually assessed and classified as non-obstructive (< 50% diameter stenosis) or obstructive (≥ 50% diameter stenosis) due to stenosis severity. The evaluation was performed by two independent experienced observers and resolved by mutual consultation. The Leiden CCTA risk score was calculated by assigning weight scores to lesion location, plaque composition, and stenosis severity. Segments without plaque were excluded, and the final score is the sum of the scores of each segment [14].

The coronary artery calcium score (CACS) is calculated by adding up the scores for calcific foci in the coronary arteries and then expressing the total calcium burden in Agatston units (AU) [15].

The severity of CCTA diameter stenosis is classified into six levels according to the Coronary Artery Disease Reporting and Data System (CAD-RADS) for predicting patients with significant CAD. CAD-RADS 0, 1, and 2 are considered non-significant CAD, while CAD-RADS 3, 4, and 5 indicate significant CAD [16].

### Data collection

Clinical data were gathered from the computerized medical files of the subjects, including age, sex, history of cardiovascular and cerebrovascular diseases, hypertension history, diabetes history, family history, smoking history, drinking history, and medication history. Laboratory tests included total cholesterol (TC), triglycerides (TG), high-density lipoprotein cholesterol (HDL-C), low-density lipoprotein cholesterol (LDL-C), fasting blood glucose (FBG), HbA1c, and serum creatinine (Cr). Estimated glomerular filtration rate (eGFR) was calculated using serum creatinine based on the chronic kidney disease epidemiology collaboration equation (CKD-EPI).

### Definition of correlative factors

The Triglyceride-Glucose (TyG) Index was calculated as log[fasting triglycerides (TG, mg/dl) × fasting glucose(FBG, mg/dl)]/2. The first group had patients with a TyG index < 7.72( divided the study population based on the median value of TyG index) and the second group had patients with a mean TyG index ≥ 7.72.

Body mass index (BMI) was calculated as weight / squared height2 (kg·m2).

Smoking was defined as currently smoking or any smoking history.

Hypertension was defined as known hypertension, antihypertensive medication use, a systolic blood pressure ≥ 140 mmHg, or diastolic blood pressure ≥ 90 mmHg.

Diabetes was defined as known diabetes or fasting plasma glucose ≥ 7.0 mmol/L.

Stroke/transient ischemic attack (TIA) was defined as a previous diagnosis of ischemic or hemorrhagic stroke or TIA.

Obstructive coronary artery disease was defined as coronary artery ≥ 50% stenosis.

### Statistics

Statistical analysis was performed using SPSS software (version 26.0; IBM SPSS Statistics). Continuous variables were presented as mean ± standard deviation or median with quartiles, and categorical variables were summarized with frequencies and percentages. Comparisons between groups were conducted by t-test or Mann-Whitney U test for continuous variables and chi-square test for categorical variables. Pearson correlation and multiple linear regression analysis coefficients were used for linear correlation between TyG index and Leiden score. Correlation analysis between TyG index and obstructive coronary artery disease was executed using univariate and multivariate logistic regression analysis.

Receiver operating characteristic (ROC) curves were used to assess the ability of the TyG index indices to predict Leiden score and Obstructive CVD.

A p-value of < 0.05 was considered statistically significant.

## Results

### Baseline characteristics of the total population

Table 1 presents a summary of the baseline characteristics of the total population across different Leiden score groups. Patients with a Leiden score ≥ 5 had significantly higher levels of age and HbA1c%, but no significant difference was observed in the TyG index among these patients.

**Table 1:** The clinical characteristics of study participants in the different Leiden score groups

	Overall (n = 427)	Leiden score < 5 (n = 138)	Leiden score ≥ 5 (n = 289)	P Value
Age (year)		78.54 ± 10.11	78.88 ± 10.39	0.39
Smoking (%)	158(37.00%)	51(36.95%)	107(37.02%)	0.26
Hypertension (%)	299(70.02%)	97(70.28%)	203(70.24%)	0.62
Dyslipidemia (%)	264(61.83)	85(61.59%)	179(61.93%)	0.58
Diabetes (%)	137(32.08%)	44(31.88%)	93(32.17%)	0.15
BMI	25.45 ± 3.38	25.58 ± 3.59	25.32 ± 3.28	0.42
Fasting blood glucose, mg/dl	110.72 ± 39.22	107.64 ± 37.44	113.76 ± 42.48	0.11
HbA1c	6.36 ± 0.88	6.05 ± 0.76	7.04 + 0.90	< 0.01
TC, mg/dl	160.869 ± 38.47	173.18 ± 38.27	168.55 ± 39.04	0.20
TG, mg/dl	128.46 (82.82, 171.88)	136.44 (83.28, 177.2)	120.49 (80.11,166.56)	0.08
LDL-C, mg/dl	93.36 ± 28.27	92.42 ± 27.45	93.58 ± 29.00	0.63
HDL-C, mg/dl	49.92 ± 14.67	51.04 ± 15.85	49.11 ± 11.98	0.08
eGFR-EPI, mL/min/1.73 m^2^	90.23(79.30, 98.42)	90.09(78.11, 99.01)	90.36(79.41, 9855)	0.78
Uric acid, umol/L	335.24 ± 76.37	331.81 ± 81.07	337.49 ± 73.29	0.44
TyG index	8.73 ± 0.62	8.77 ± 0.65	8.71 ± 0.57	0.55
Statins use (%)	262(61.35%)	83(60.14)	179(61.93)	0.22
Ezetimibe use (%)	18(42.15%)	6(4.34)	12(4.15)	0.71

**Notes**: TyG: Triglyceride- Glucose Index; BMI: body mass index; TC: Total cholesterol; TG: triglyceride; LDL-C: Low-density lipoprotein cholesterol; HDL-C: high-density lipoprotein cholesterol; HbA1c: glycated hemoglobin; Cr: creatinine. P values < 0.05 show statistically significant differences

On the other hand, Table 2 summarizes the baseline characteristics of the total population across different TyG index tertiles. Patients with a TyG index ≥ 7.72 displayed significantly higher levels of Diabetes, BMI, HbA1c, TC, and TG, Leiden score and lower levels of HDL-C.

**Table 2:** The clinical characteristics of study participants in the different TyG index tertiles

	Overall (n = 427)	TyG index tertiles < 7.72 (n = 213)	TyG index tertiles ≥ 7.72 (n = 214)	P Value
Age (year)	79.66 ± 9.26	79.66 ± 9.26	77.82 ± 10.30	0.13
Smoking (%)158	158(37.00%)	79(37.09%)	79(36.91%)	0.35
Hypertension (%)299	299(70.02%)	151(70.89%)	148(69.15%)	0.40
Dyslipidemia (%)	264(61.83)	129(60.56)	135(63.08)	0.66
Diabetes (%)137	137(32.08%)	60(28.16%)	77(35.98%)	0.02
BMI	25.45 ± 3.38	24.36 ± 2.96	26.12 ± 344	< 0.001
Fasting blood glucose, mg/dl	110.72 ± 39.22	96.30 + 20.70	116.28 ± 29.83	< 0.001
HbA1c	6.36 ± 0.88	6.32 ± 0.85	6.48 + 1.12	0.28
TC, mg/dl	160.869 ± 38.47	163.08 ± 40	172.31 ± 36.15	0.05
TG, mg/dl	128.46 (82.82, 171.88)	120.49 (83.28,166.56)	310.1 (83.28, 177.2)	< 0.01
LDL-C, mg/dl	93.36 ± 28.27	89.78 ± 29.16	92.85 ± 27.24	0.38
HDL-C, mg/dl	49.92 ± 14.67	52.56 ± 13.81	45.27 ± 11.12	< 0.01
eGFR-EPI, mL/min/1.73 m^2^	90.23(79.30, 98.42)	90.10(79.41, 98.24)	90.37(79.22, 98.59)	0.72
Uric acid, umol/L	335.24 ± 76.37	350.55 ± 77.43	327.96 ± 75.19	0.01
Leiden score	10.69 ± 0.75	9.82 ± 0.62	11.58 ± 0.85	0.07
Statins use (%)262	262(61.35%)	246(61.50)	16(59.25)	0.13
Ezetimibe use (%)18	18(42.15%)	10(4.69)	8(3.73)	0.39

**Notes**: TyG: Triglyceride- Glucose Index; BMI: body mass index; TC: Total cholesterol; TG: triglyceride; LDL-C: Low-density lipoprotein cholesterol; HDL-C: high-density lipoprotein cholesterol; HbA1c: glycated hemoglobin; Cr: creatinine. P values < 0.05 show statistically significant differences

According to CAD-RADS, 228(53.39%) were categorized as non-significant CAD and 199(46.60%) as significant CAD as presented in Table 3. There were no significant differences in high TyG index group than that in the low TyG index group (Non-significant CAD (%): 53.99% vs. 52.80%, p = 0.78; Significant CAD (%):46.01% vs. 47.19%, p = 0.86). The Leiden score was similar in the high TyG index group and in the low TyG index group (9.82 ± 0.62 vs. 11.58 ± 0.85, p = 0.42) (Table 3). And the overall median CACS in the high TyG index group was also similar with in the low TyG index group [89.7(0, 641.7) vs. 88.51(35.8, 621.2), P < 0.001].

**Table 3:** Coronary computed tomography angiography findings in the various groups

	TyG index tertiles < 9 (n = 213)	TyG index tertiles ≥ 9 (n = 214)	P Value
Non-significant CAD (%)			
CAD-RADS 0, 1, 2	115(53.99%)	113(52.80%)	0.78
Significant CAD (%)			
CAD-RADS 3, 4, 5	98(46.01%)	101(47.19%)	0.86
CACS	89.7(0, 641.7)	88.51(35.8, 621.2)	0.58
Leiden score	9.82 ± 0.62	11.58 ± 0.85	0.42

**Notes**: CACS: Coronary artery calcification scoring; CAD-RADS Score: Coronary Artery disease-reporting and data system score. CAD-RADS 0, 1, and 2 are considered non-significant CAD, while CAD-RADS 3, 4, and 5 indicate significant CAD. P values < 0.05 show statistically significant differences

### Pearson correlation and multiple linear regression analysis for the association between TyG index and leiden score

**Fig. 1:**
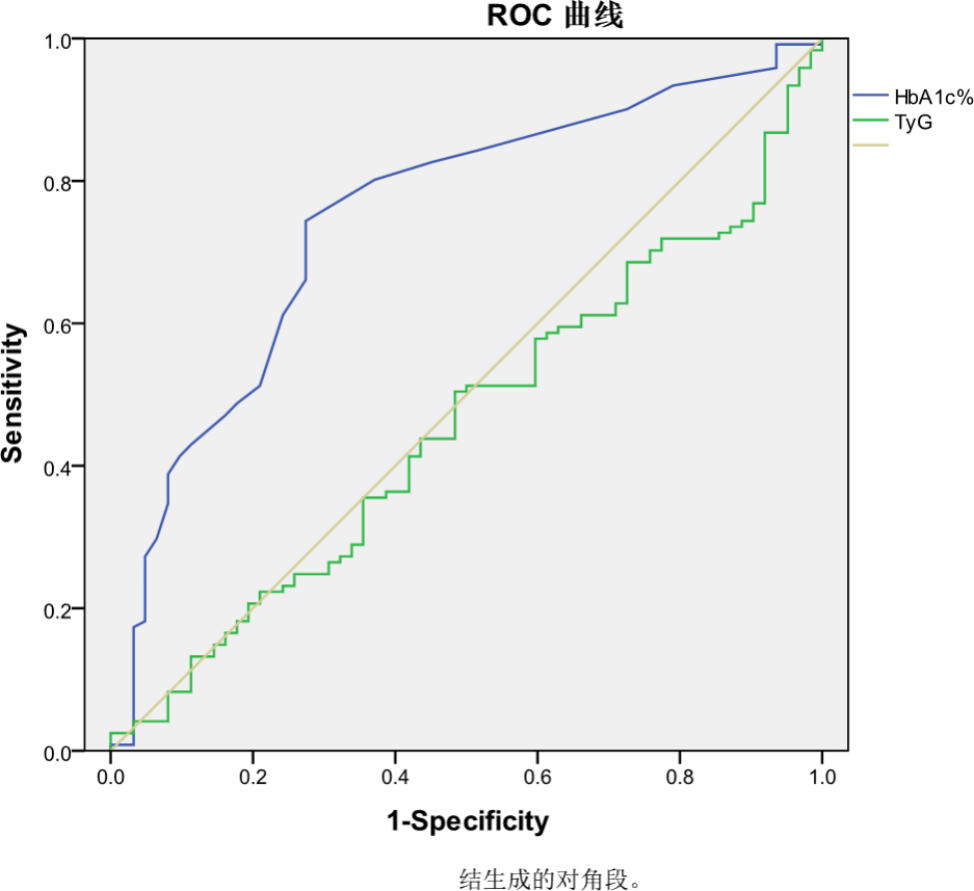
displays the ROC curve of the Leiden score, with an AUC of 0.458 (95% confidence interval: 0.372–0.544, P = 0.34) for the TyG index and an AUC of 0.748 (95% confidence interval: 0.673–0.824, P < 0.01) for HbA1c%

The Pearson correlation analysis showed that HbA1c% (r = 0.436, p < 0.001), age (r = 0.34, p < 0.01), and FBG (r = 0.153, p = 0.013) had positive correlations with the Leiden score (Table 4). Conversely, no correlation was observed between the TyG index and the Leiden score (r = 0.050, p = 0.415). Multiple linear regression analysis further confirmed that HbA1c% (β = 3.418, 95% confidence interval [CI]: 2.215–4.621, P < 0.001) was positively correlated with the Leiden score, while no significant correlation was observed between the TyG index and the Leiden score (β = 0.714, 95% CI: -7.158- 5.012, P = 0.714). In terms of predicting the occurrence of Leiden score, the maximum area under the ROC curve was 0.748 (95% CI: 0.673–0.824) for HbA1c% and 0.458 (95% CI: 0.372–0.544) for TyG index (Fig. 1).

**Table 4:** Pearson correlation and multiple linear regression analysis for the association between TyG and Leiden score

Characteristic	Pearson		Multivariable	
	r	P Value	β	P Value
TyG	0.050	0.415	0.714(-7.158- 5.012)	0.714
age(years)	0.252	< 0.001	0.1579(0.033–0.280)	0.013
BMI	0.001	0.996	-0.033(-0.411-0.346)	0.865
TC, mg/dl	0.079	0.087	--	--
TG, mg/dl	0.082	0.075	--	--
LDL-C, mg/dl	0.017	0.719	--	--
HDL-C, mg/dl	-0.067	0.147	--	--
HbA1c%	0.436	< 0.001	3.418(2.215–4.621)	< 0.001
Fasting blood glucose, mg/dl	0.153	0.013	0.715(-2.006-3.435)	0.605
eGFR-EPI, mL/min/1.73 m2	-0.145	0.002	3.418(-0.126-7.553)	0.161

**Notes**: OR: odds ratio; TyG: Triglyceride- Glucose Index; BMI: body mass index; TC: Total cholesterol; TG: triglyceride; LDL-C: Low-density lipoprotein cholesterol; HDL-C: high-density lipoprotein cholesterol; HbA1c: glycated hemoglobin; Cr: creatinine. P values < 0.05 show statistically significant differences

### Univariable and multiple logistic regression analysis for the association between TyG index and obstructive CVD

**Fig. 2:**
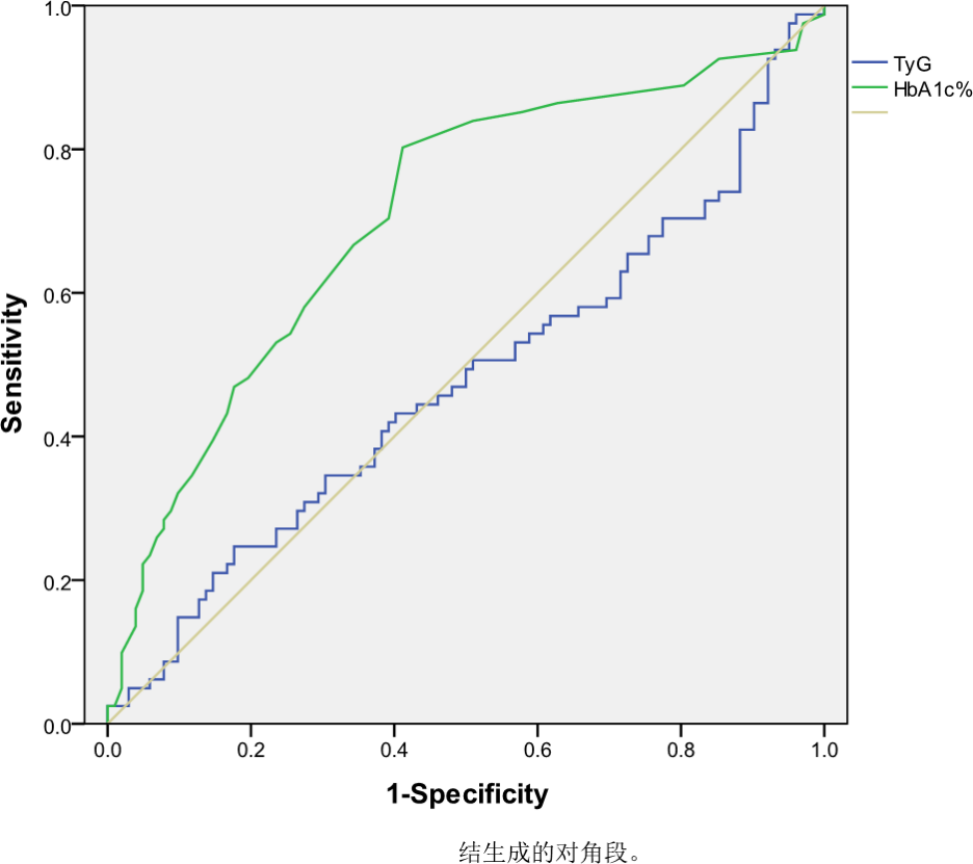
shows the ROC curve of Obstructive CVD, with an AUC of 0.477 (95% confidence interval: 0.390–0.563, P = 0.58) for the TyG index and an AUC of 0.708 (95% confidence interval: 0.631–0.785, P < 0.01) for HbA1c%

Furthermore, the analysis results indicated that HbA1c% was an influential factor for obstructive CVD (β = 1.296, 95% CI: 1.109–1.514, P < 0.001), while the TyG index was not an independent risk factor for obstructive CVD (β = 1.184, 95% CI: 0.761–1.842, P = 0.454) (Table 5). The maximum area under the ROC curve to predict the occurrence of obstructive CVD was 0.708 (95% CI: 0.631–0.785) for HbA1c% and 0.477 (95% CI: 0.390–0.563) for the TyG index (Fig. 2).

**Table 5:** Univariable and Multiple logistic regression analysis for the association between TyG and Obstructive CVD

	Univariate analysis		Multivariate analysis	
	HR (99% CI)	P-value	HR (99% CI)	P-value
TyG	1.001(0.927–1.081)	0.981	1.184(0.761–1.842)	0.454
Age(years)	1.036(1.016–1.056)	< 0.001	1.036(0.998–1.075)	0.064
BMI	0.989(0.937–1.045)	0.700	--	--
TC, mmol/L	0.811(0.674–0.976)	0.026	--	--
TG, mmol/L	0.795(0.641–0.987)	0.037	--	--
LDL-C, mmol/L	0.278(0.871–1.118)	0.278	--	--
HDL-C, mmol/L	0.654(0.380–1.125)	0.125	--	--
HbA1c%	1.296(1.109–1.514)	< 0.001	2.686(1.071–6.730)	0.035
Fasting glucose, mmol/L	0.874(0.720–1.061)	0.001	--	--
eGFR-EPI, mL/min/1.73 m^2^	1.009(0.999–1.019)	0.069	--	--
Smoke	1.927(1.155–3.214)	0.012	1.600(0.615–4.164)	0.336
Hypertension	1.575(0.942–2.635)	0.084	--	--
DIabetes	1.383(0.839–2.281)	0.203	--	--
Statins use	1.753(1.184–2.597)	0.005	1.328(0.457–3.862)	0.602
Ezetimibe use	4.243(1.500-12.005)	0.006	0.732(0.106–5.035)	0.751

## Discussion

This is the first study to observe the relationship between levels of TyG and the Leiden CCTA risk score in elderly male patients. In the present elderly male study, the TyG index was found to have no significant association with the Leiden score and obstructive CVD as a risk factor, while HbA1c% was found to be an influential factor for Leiden score and obstructive CVD. Further large-scale prospective studies are warranted to determine the predictive value of the TyG index for cardiovascular health in elderly patients.

Sufficient evidence has shown that the TyG index, a reliable alternative marker of insulin resistance (IR), can predict the cardiovascular prognosis of CVD patients [17]. The TyG index has been gradually linked to the development of acute coronary syndrome (ACS), atherosclerosis, heart failure, and coronary artery calcification [18]. Several potential mechanisms support TyG as a biomarker of CVD, including the role of metabolic flexibility, endothelial dysfunction, coagulation disorders, and smooth muscle cell dysfunction [19]. As a first-line non-invasive imaging technique, CCTA is widely used in patient evaluation and follow-up for CAD [20]. The application of CCTA can not only realize the qualitative evaluation of plaque extent, location, severity, and high-risk plaque characteristics but also reflect the severity of coronary artery disease through a variety of quantitative scores, such as Leiden score. The Leiden CCTA risk score, incorporating coronary plaque extent, location, severity, and composition, provides precise prediction of future events and superior risk stratification than a score based on stenosis severity only [14].

In this study, discordance analyses helped us understand the consequences of the TyG index via the disagreements between the TyG index and HbA1C. Unlike previous studies, the present study investigated the relationship between TyG index and the Leiden CCTA risk score in an elderly male population. In the present study, we found no association between TyG and the Leiden score; however, we identified an association with HbA1c and Leiden score. These differences may be attributed to the fact that the subjects of the study are all male. Some trials have highlighted the difference between the TyG index and the early stages of subclinical atherosclerosis between the sexes. The RCSCD-TCM study in China showed a weaker association between the TyG index and CAP in male CHD than in females [21]. All insulin replacement markers showed a stronger association with HOMA-IR between females than that of males [22]. The ILAS Study (I-Lan Longitudinal Aging Study) [23] showed that the TyG index was significantly associated with carotid intima-media thickness (cIMT) in women but not in men.

In addition, it is necessary to exercise caution when predicting cardiovascular events in the elderly due to the obvious degradation of physiological functions. While the TyG index is a useful marker for predicting the risk of CVD development and progression, its ability to discriminate CVD in elderly individuals remains to be explored. The RCSCD-TCM study conducted in China found that the association between the TyG index and CAP in middle-aged patients is higher than that in elderly patients [21]. This decline in the impact of metabolic risk factors on diabetes in the elderly may be partially explained by the biological effect of aging on glucose metabolism, such as the decline in insulin secretion, β-cell sensitivity, and acute insulin response to glucose with age [24]. Additionally, anabolism is significantly lower in elderly patients compared to middle-aged-onset patients. To improve the accuracy of predicting cardiovascular events in the elderly, the TyG index should be used in conjunction with other indicators such as serum creatinine and C-reactive protein.

There are several limitations related to this report. Firstly, the present study was retrospective, and a prospective cohort study may help reduce selection bias. Secondly, this study mainly included middle-aged and elderly patients, since they were more likely to develop coronary atherosclerosis. Finally, the number of patients with more than twice CCTA examinations without receiving reperfusion therapy was quite small, and a larger sample size research is needed.

In conclusion, the study revealed that the TyG index did not show any significant correlation with the Leiden score and obstructive CVD as a risk factor in elderly male population. On the other hand, HbA1c% was identified as an influential factor for both the Leiden score and obstructive CVD.

## Data Availability

The datasets used or analysed during the current study are available from the corresponding author on reasonable request.

## Abbreviations

ACS: Acute coronary syndrome
AU: Agatston units
BMI: Body Mass Index
cIMT: Carotid intima-media thickness
CCTA: Coronary Computed Tomography Angiogram
CAD: Coronary artery disease
CHD: Coronary heart disease
CAP: Carotid artery plaque
CACS: The coronary artery calcium score CACS
CAD-RADS: Coronary Artery Disease Reporting and Data System
TC: Total cholesterol
TG: Triglycerides
LDL-C: Low -density lipoprotein cholesterol
HDL-C: High -density lipoprotein cholesterol
FBG: Fasting blood glucose
eGFR: Estimated glomerular filtration rate
CKD-EPI: Chronic kidney disease epidemiology collaboration equation
TIA: Transient ischemic attack
TyG: Triglyceride-glucose
